# Supplementary material for: The Gut Microbiota May Affect Personality in Mongolian Gerbils
Source: Microorganisms. 2022 May 20;10(5):1054. doi: 10.3390/microorganisms10051054 (PMC9146877; doi:10.3390/microorganisms10051054)
Supplement: Supplementary file 1 [file microorganisms-10-01054-s001.zip › microorganisms-1673295-supplementary.pdf]

Supplemental Material

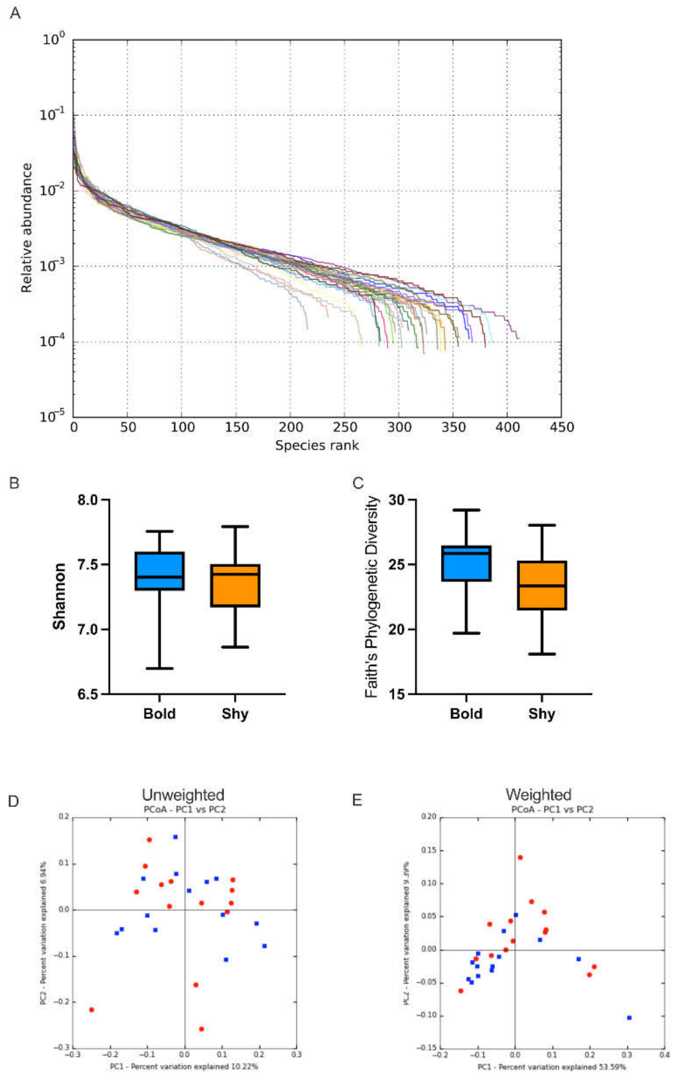

Figure S1. Alpha and beta diversity of gut microbiota in gerbils. (**A**) Rank Abundance of ASVs. (**B**) Alpha diversity: Shannon index. (**C**) Alpha diversity: Faith's phylogenetic diversity. (**D**) PCoA plot of unweighted UniFrac distance metrics. (**E**) PCoA plot of weighted UniFrac distance metrics.
